# Dilated thoracoabdominal and epigastric veins in a hemodialysis patient with SVC occlusion: case report and literature review

**DOI:** 10.3389/fcvm.2025.1645455

**Published:** 2025-07-18

**Authors:** Yanlin Zhou, Bo Tu, Ziming Wan

**Affiliations:** ^1^ Department of Nephrology, Metabolism and Immunology Laboratory for Urological Diseases, The First Affiliated Hospital of Chongqing Medical University, Chongqing, China; ^2^ Department of Ultrasonography, The First Affiliated Hospital of Chongqing Medical University, Chongqing, China

**Keywords:** central venous stenosis, superior vena cava occlusion, hemodialysis, arteriovenous fistula, endovascular treatment

## Abstract

**Introduction:**

For several reasons, the incidence of superior vena cava(SVC) obstruction continues to rise, as a serious complication of hemodialysis(HD) access, and is becoming a major cause of access depletion. It is also the most difficult challenge for vascular access workers. Here we present the case of a HD patient with complete SVC occlusion, and why no intervention was made.

**Case presentation:**

A 50-year-old man on maintenance HD was admitted for markedly dilated thoracoabdominal wall veins and superficial epigastric veins. Digital subtraction angiography(DSA) revealed a complete occlusion of the SVC. Treatment options include interventional therapy, closing the arteriovenous fistula(AVF) to reduce venous pressure and creating a new lower extremity arteriovenous(AV) access, or open surgery. The patient's venous hypertension syndrome and AV access function were carefully evaluated, leading to a decision for conservative management without immediate intervention. After five years of follow-up, his left forearm AVF continues to function well, and both the AVF and superficial epigastric veins can be used for HD access.

**Conclusion:**

The management of central venous stenosis(CVS)/obstruction continues to present significant challenges. Presently, endovascular treatment is associated with low primary patency rates and a high risk of complications. Patient-centered decision-making plays a crucial role in the management of CVS/obstruction.This study provides significant insights into the conservative management in complete SVC occlusion, characterized by comparable excellent collateral compensation.

## Introduction

The SVC is the largest vein, conveying blood from the head, neck, arm, and chest to the right atrium. Partial or complete obstruction the SVC is observed most frequently in lung cancer and lymphoma. With the increase in non-malignant diseases, dialysis catheters now account for about 5% of SVC obstruction (1). SVC stenosis occurs in approximately 9.4% of chronic HD patients carrying a tunneled cuffed catheter (2).

Two primary effects of SVC obstruction on maintenance HD patients are observed (3, 4). The first is venous hypertension syndrome, manifesting with limb swelling or pain, cutaneous congestion, pigmentation, or even ulceration, and open superficial chest wall veins. If the SVC lesions are serious, facial edema, head and neck distention, pleural effusion, and chylothorax may be observed. Another manifestation is access dysfunction, including decline of HD adequacy, increase of venous pressure during HD, prolonged hemostasis after needle removal from AVF, continuous progress of fistula tumor-like dilatation, and repeated thrombosis in the fistula.

Patients with HD have fewer symptoms of SVC obstruction compared with cancer patients (5), perhaps because HD patients may have a longer sub-clinical or chronic process leading to SVC occlusion, providing time for collateral circulation development (6). Here we report the case of a 50-year-old man receiving HD who was admitted for markedly dilated thoracoabdominal wall and superficial epigastric veins, and discuss the therapeutic strategy used.

## Case report

**Figure 1:**
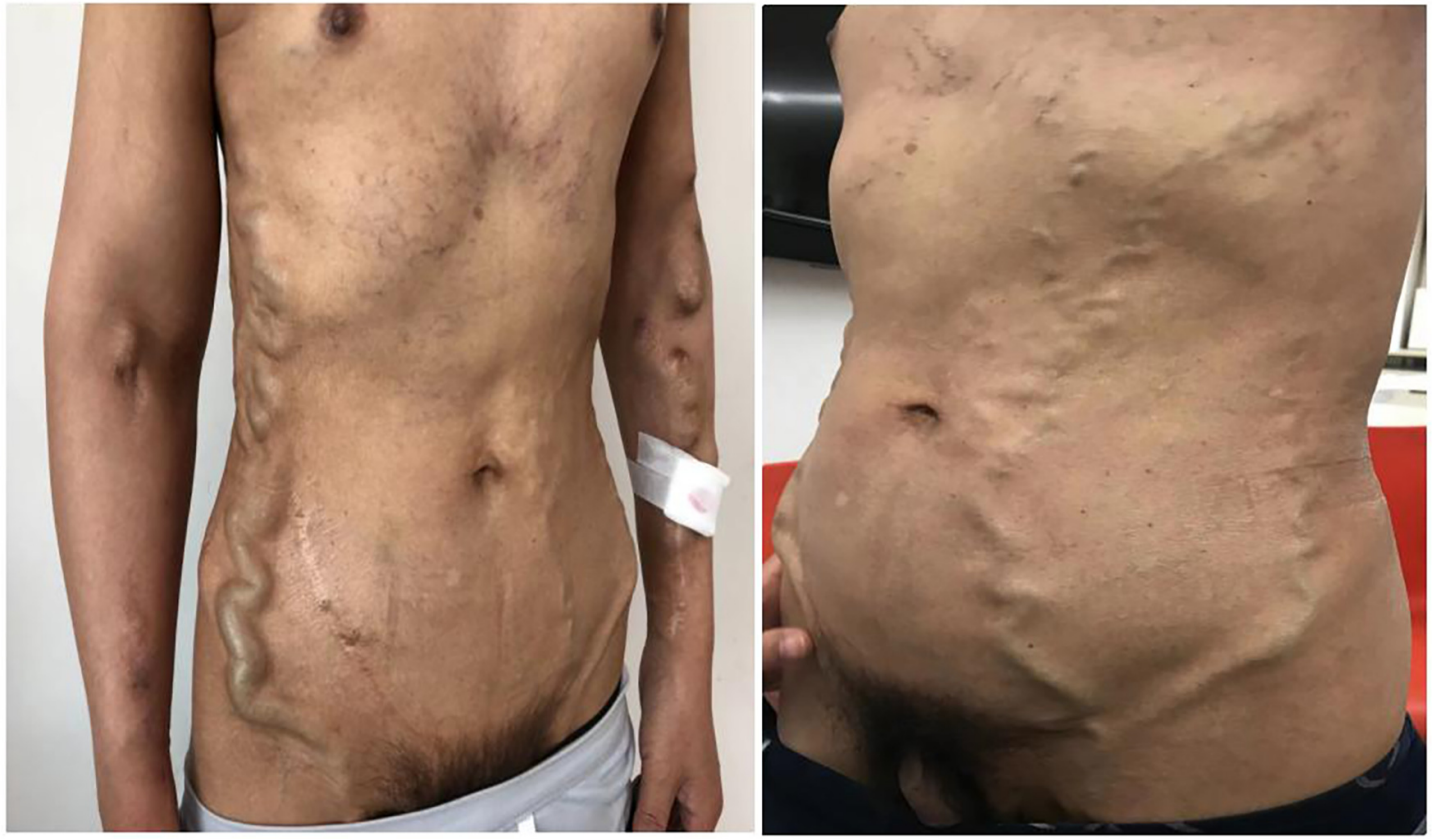
The patient presented markedly dilated thoracoabdominal wall and superficial epigastric veins.

A 50-year-old man maintained with HD was admitted for markedly dilated thoracoabdominal wall and superficial epigastric veins (Figure 1), with notable machinery murmur and tremor. He had previously been diagnosed with uremia in 1994, and a right forearm AVF was established for HD access. He received kidney transplantation in 1995, but the renal allograft failed in 2007, necessitating further dialysis. He had a semi-permanent catheter placed in the right internal jugular vein from 2011 to 2014 following an occluded right forearm and right elbow AVF. A left forearm AVF was established in 2015 and used for HD access until the present illness. The patient's complete vascular access history was presented in Table 1.

**Table 1:** Vascular access history of the patient.

Year	Vascular access
1994–1995	Direct puncture of the radial artery and cephalic vein, and a right forearm AVF was established, then he continuted HD with mature AVF.
2007–2011	He continued dialysis with normal function of the right forearm AVF.
2011	The right forearm AVF was occluded, then the right elbow AVF was reconstructed and subsequently occluded.
2011–2014	A semi-permanent catheter was placed in the right internal jugular vein.
2014–2015	The blood flow in the right internal jugular vein semi-permanent catheter remained poor despite repeated administration of urokinase thrombolysis and replacement with a semi-permanent catheter.
2015-until now	A left forearm AVF was successfully established and matured for use in the HD unit until now, after which the right internal jugular vein semi-permanent catheter was removed.

**Figure 2:**
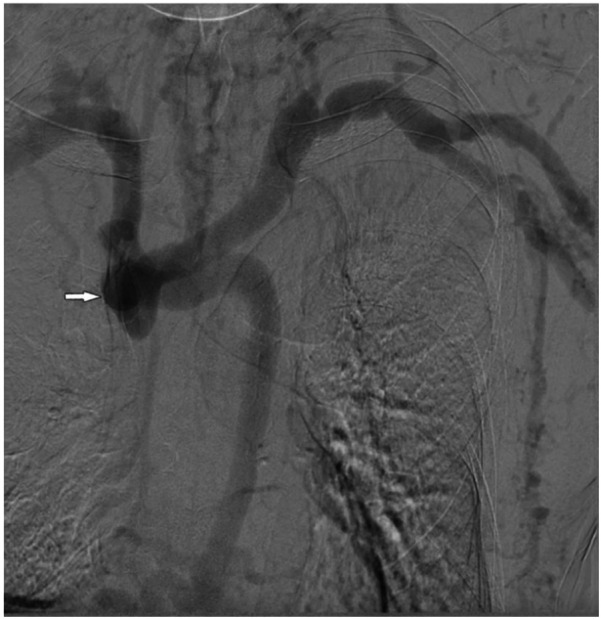
DSA revealed a complete occlusion of the SVC.

At presentation, vital signs were within normal limits, and there was no swelling or pain in the extremities, neck, or head. Cutaneous examination revealed no congestion or ulceration, infection, nonhealing wounds, or incisions. He also reported no symptoms of the respiratory (hoarse voice, dyspnea) or neurological (visual or auditory disturbances, cognitive disabilities) systems. Blood cell count and liver function were normal. Abdomen ultrasound did not identify cirrhosis, splenomegaly, or portal vein thrombosis. Thoracic computed tomography showed no abnormalities of lung or mediastinum and no pleural effusion. Echocardiography showed no abnormal cardiac structure or function. DSA revealed a complete occlusion of the SVC (Figure 2) and a markedly dilated azygos vein. Vascular access function was further assessed, and color Doppler ultrasound of the AVF identified no fistula stenosis. During multiple dialysis sessions, no increased venous pressure was observed, and there was no prolonged hemostasis after HD. Dialysis adequacy was measured by single pool Kt/V(SpKt/V). The calculated SpKt/V was >1.2. Based on these observations, the patient was determined to have a complete SVC occlusion with markedly dilated thoracoabdominal wall and superficial epigastric veins, but no other venous hypertension symptoms and signs, and no evidence of access dysfunction. After thoroughly discussing the treatment options and associated risks with the patient, a conservative management approach was opted without immediate intervention, but following him closely. Every month, we recorded the appearance, diameter, and size of the lateral branches. We carefully examined whether the patient developed any new related symptoms and signs of SVC occlusion. Every three months, SpKt/V was used to evaluate HD adequacy, and the AVF was monitored by ultrasound.

**Figure 3:**
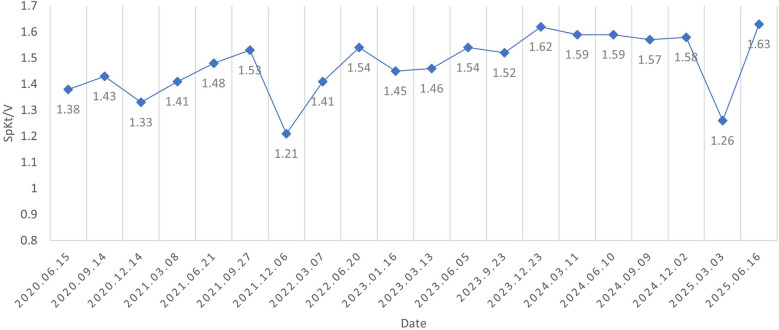
SpKt/V was assessed every three months and remained above 1.2 throughout the five-year follow-up period.

**Figure 4:**
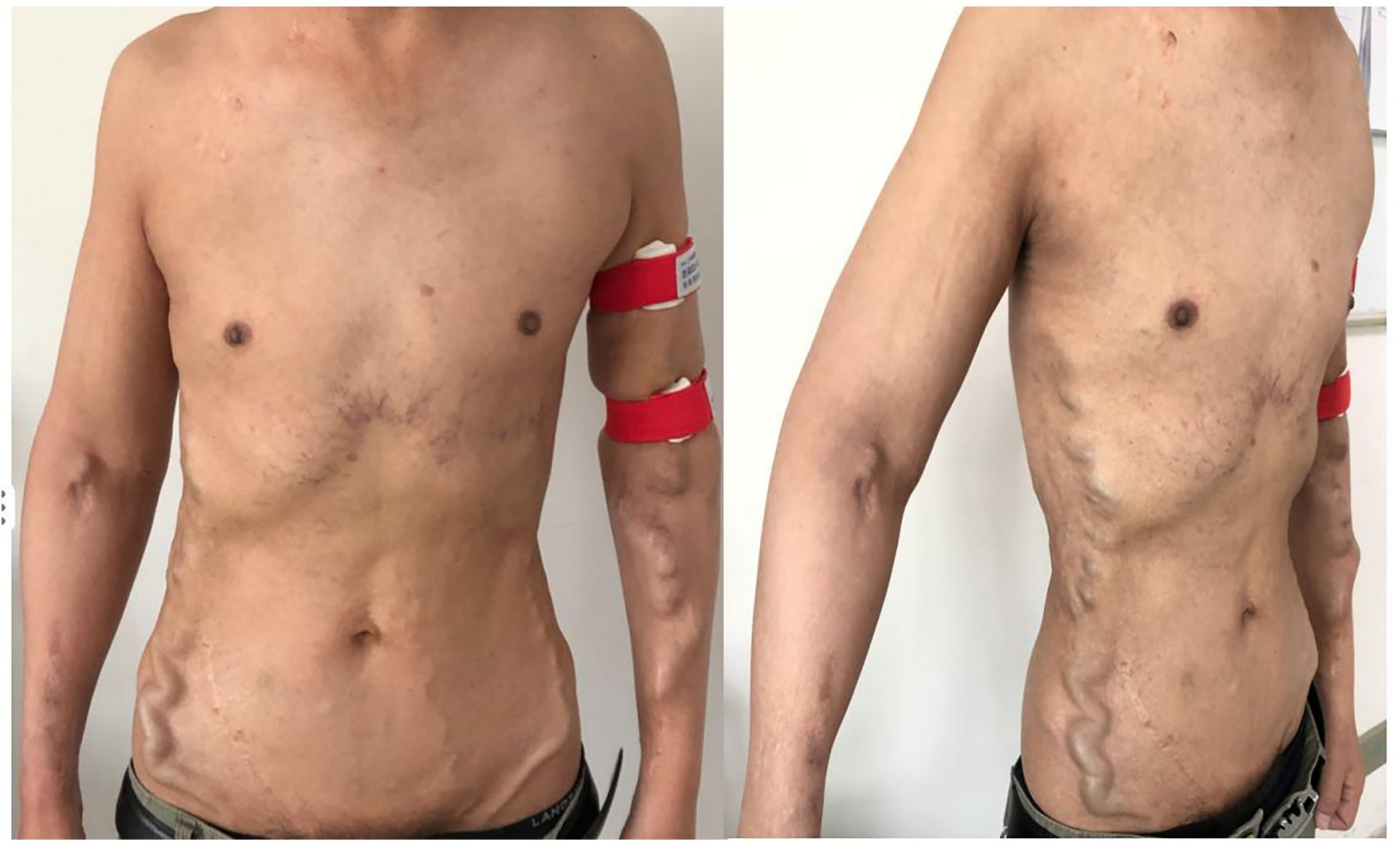
The appearance of collateral brancheswas comparable to that observed five years ago.

After five years of follow-up, HD sufficiency was evaluated every 3 months. SpKt/V remained acceptable; we here present 5 years of data (Figure 3). His left forearm AVF continues to function well. Both the AVF and compensated superficial epigastric veins could be punctured for HD access. Moreover, the dilated thoracoabdominal wall veins and superficial epigastric veins remained unchanged (Figure 4). No new symptoms or signs of SVC obstruction or serious complications, including gastrointestinal and intracranial disease, developed. We continue to follow him conservatively.

## Discussion

Most HD patients have only mild or no symptoms, and the central veins are not routinely imaged. The incidence of CVS/obstruction in publications ranged from 3% to 60% (7), but the incidence of complete SVC obstruction is very low. Non-tunneling catheter use, subclavian vein or left jugular vein access, repeated catheterization, and prolonged indwelling time all increase the risk of CVS/occlusion (8). The patient's SVC obstruction was related to the long indwelling time of the semi-permanent catheter in the right internal jugular vein.

It is vital to choose an appropriate therapeutic schedule for a patient with SVC obstruction. Treatment options (9) for SVC obstruction include interventional therapy, closing the AVF to reduce the venous pressure of compensatory vessels with the need to create a new, lower extremity AV access, and open surgery. Due to the numerous underlying complications in HD patients, the difficulty and risk of anesthesia with surgical procedures are very high. Endovascular therapy, including percutaneous transluminal angioplasty (PTA) or PTA with stenting, is currently the preferred treatment for SVC occlusions because it is minimally invasive (10). Surgical operations should be considered secondary and are only used when endovascular therapy has failed. These interventions succeed in opening the lesion in 40%–80% of cases. However, primary patency with PTA or PTA with stenting for CVS/occlusions is very poor, about 82.3% and 88.9% at one month, 57.1% and 67.8% at 6 months, with 1-year rates of 38.9% and 53.8%, 2-year rates of 29.4% and 39.4%, respectively (11). Zhao YL et al. (12) reported that the overall 2-year vascular access patency rate following PTA with stenting for SVC stenosis in HD patients was 33.2%. Moreover, SVC obstruction in HD patients often includes thrombosis, fibroplasia, calcification, and even long-segment occlusion, and a standard wire may not be able to cross the lesion. More aggressive techniques, such as sharp recanalization and radiofrequency guidewire, may be required to transverse the blockage (7). The most serious complications that can occur during central venous recanalization include the perforation of large veins, leading to massive hemorrhage in the chest or pericardial space, pericardial tamponade, acute heart failure, and pneumothorax (13). If endovascular therapy for SVC obstruction is successful, venous hypertension is reduced and the degree of collateral branches is visibly decreased. However, opening a completely obstructed SVC is difficult and high-risk, and repeated intervention due to low patency may be required.

Endovascular treatment for asymptomatic CVS can cause more rapid progression to symptomatic CVS/occlusion (14). The 2019 update to the KDOQI Clinical Practice Guideline for Vascular Access (15) recommended that CVS/occlusion does not require intervention in asymptomatic or mildly symptomatic patients with working HD access. This was the case for our patient with very severe, complete SVC occlusion and excellent collateral compensation without swelling, pain, respiratory or neurologic symptoms, or access dysfunction. Whether to perform an intervention warrants careful evaluation. The esophageal varices are observed commonly in HD patients with SVC obstruction. About 8% of patients with these varices may have upper gastrointestinal bleeds, and some can experience major hemorrhage (5, 16). We recommended that this patient undergo gastroscopy to evaluate the presence of esophageal varices, which was at risk of future massive gastrointestinal bleeding. Unfortunately, our patient refused gastroscopy. Intracranial hypertension can present with non-specific signs and symptoms that are not easily detected. Cases have been reported of CVS/occlusion in HD patients leading to severe neurological diseases, including idiopathic perimesencephalic subarachnoid hemorrhage, cerebral infarction, venous congestive encephalopathy, epilepsy, and syncope (17). Rupture and bleeding of lateral branches, as well as thrombosis, pose serious risks to the patient and may potentially endanger his life.

Conservative treatment was selected after communicating treatment options to the patient, given the potential and serious risks for the patient. Venous hypertension remains a risk, so clinical symptoms and signs must be closely observed, and access function regularly monitored. Gastroscopy and neuroimaging should be performed to further evaluation upon patient consent.The patient was followed up at regular intervals of one to three months throughout the five-year follow-up period and remains clinically stable. Conservative treatment appears to have been beneficial for this patient. However, further clinical studies are needed to confirm this observation. The natural history and underlying mechanisms of interaction between CVS/obstruction and collateral circulation merit further study.

## Data Availability

The original contributions presented in the study are included in the article/Supplementary Material, further inquiries can be directed to the corresponding author.
